# Clinical outcomes and actual consequence of lung nodules incidentally detected on chest radiographs by artificial intelligence

**DOI:** 10.1038/s41598-023-47194-6

**Published:** 2023-11-13

**Authors:** Shin Hye Hwang, Hyun Joo Shin, Eun-Kyung Kim, Eun Hye Lee, Minwook Lee

**Affiliations:** 1 Department of Radiology, Research Institute of Radiological Science and Center for Clinical Imaging Data Science, Yongin Severance Hospital, Yonsei University College of Medicine, 363, Dongbaekjukjeon-daero, Giheung-gu, Yongin-si, Gyeonggi-do 16995 Republic of Korea; 2 Center for Digital Health, Yongin Severance Hospital, Yonsei University College of Medicine, Yongin-si, Gyeonggi‑do Republic of Korea; 3 Division of Pulmonology, Allergy and Critical Care Medicine, Department of Internal Medicine, Yongin Severance Hospital, Yonsei University College of Medicine, Yongin-si, Gyeonggi-do Republic of Korea

**Keywords:** Medical research, Cancer, Respiratory tract diseases

## Abstract

This study evaluated how often clinically significant lung nodules were detected unexpectedly on chest radiographs (CXR) by artificial intelligence (AI)—based detection software, and whether co-existing findings can aid in differential diagnosis of lung nodules. Patients (> 18 years old) with AI-detected lung nodules at their first visit from March 2021 to February 2022, except for those in the pulmonology or thoracic surgery departments, were retrospectively included. Three radiologists categorized nodules into malignancy, active inflammation, post-inflammatory sequelae, or “other” groups. Characteristics of the nodule and abnormality scores of co-existing lung lesions were compared. Approximately 1% of patients (152/14,563) had unexpected lung nodules. Among 73 patients with follow-up exams, 69.9% had true positive nodules. Increased abnormality scores for nodules were significantly associated with malignancy (odds ratio [OR] 1.076, *P* = 0.001). Increased abnormality scores for consolidation (OR 1.033, *P* = 0.040) and pleural effusion (OR 1.025, *P* = 0.041) were significantly correlated with active inflammation–type nodules. Abnormality scores for fibrosis (OR 1.036, *P* = 0.013) and nodules (OR 0.940, *P* = 0.001) were significantly associated with post-inflammatory sequelae categorization. AI-based lesion-detection software of CXRs in daily practice can help identify clinically significant incidental lung nodules, and referring accompanying lung lesions may help classify the nodule.

## Introduction

Due to advances in artificial intelligence (AI) applications in radiology, several AI-based lesion-detection software programs have been introduced for chest radiographs (CXRs)^1^ idd. Excellent performance has been reported in the detection of major chest abnormalities, including lung nodules^2–8^. As these reports were derived from a disease-enriched experimental dataset^9,10^, and the performance of diagnostic tests may vary depending on the characteristics of the population and disease prevalence^11^, their performance should be verified in multiple cohorts. The ability of AI to successfully detect lung nodules has been verified in the real world using emergency department records^12^, lung cancer screening^13–16^, and respiratory outpatient clinic cohorts^17^.

However, few studies have evaluated the clinical implications of AI detection of unexpected lung nodule in patients whose initial concern was not chest disease. Furthermore, as AI-based detection is approved only as an auxiliary tool to help doctors detect abnormalities in CXRs^18^, performance of computed tomography (CT) scans of the detected lung nodules depends on the judgment of the physician, who relies on clinical information such as patient symptoms, risk factors, past history, and blood tests. In addition, there is concern about the likelihood of increased false positive results when using AI to detect lung nodules. The number of clinically meaningful lung nodules, such as suspicion of malignancy or active infection, necessitating further investigations or interventions, among those unexpectedly detected by AI and whether using AI software can change patient management are important and unresolved questions.

One of the difficulties in interpreting CXR is that different pathologies may present similar imaging findings. Because CXR is a two-dimensional projection of a three-dimensional lesion, radiologic features that allow for differentiation among pathologies in cross-sectional images may not be evident. In classic radiologic interpretation, a differential diagnosis is based on comprehensive evaluation of the presence of major co-existing radiologic findings (e.g., nodules, fibrosis, consolidation, and pleural effusion), their distribution, and the characteristics of the main lesion. Similarly, lung nodules with different pathologies can be detected by AI using CXR, and differential diagnosis can be attempted using co-existing radiographic findings^19^.

Therefore, the purpose of this study was to evaluate how often clinically significant lung nodules were detected unexpectedly on CXR, assesses how patient management is influenced by use of AI software, and determines whether the co-existing findings by AI can aid in the differential diagnosis of lung nodules on CXR.

## Materials and methods

The institutional review board of our institution approved this retrospective study (Institutional Review Board, Yongin Severance Hospital, Yonsei University College of Medicine: 9-2022-0070) and waived the requirement for informed consent. The study was carried out in accordance with the Declaration of Helsinki.

### Inclusion and exclusion criteria

Patients (> 18 years old) who underwent CXR in posteroanterior (PA) and anteroposterior (AP) views during their first visit to an outpatient clinic in our hospital and in whom a lung nodule was unexpectedly detected by AI were included in the study. We excluded patients who had visited a pulmonology or thoracic surgery department due to the possibility of intrathoracic problems. We also excluded patients who did not receive a follow-up CXR or CT scan after nodule detection to minimize inconclusive results. For the same reason, we excluded patients without final clinical diagnosis concerning lung nodules, such as suspicion of malignancy or active infection or cases requiring further investigations or interventions, at follow-up by reviewing electronic medical records (EMRs).

### Lung nodule detection by AI software on CXR

In our hospital, commercially available AI-based lesion-detection software (Lunit INSIGHT CXR, version 3, Lunit Inc., Republic of Korea) was applied to all CXRs with PA and AP views since March 2021. The software could detect eight varieties of lesions, including nodule, pneumothorax, consolidation, atelectasis, fibrosis, cardiomegaly, pleural effusion, and pneumoperitoneum, with a contour map for localization^20^. Lesions were considered present when the abnormality score exceeded 15%^9,21,22^. When the patient underwent CXR, the analyzed AI result was automatically attached to the original image as a secondary file in the picture archiving and communication system (PACS). Doctors could refer to the AI results about lung nodules displayed as a contour map, abbreviation, and abnormality score when assessing original radiographs. This allows for real-time utilization of AI-generated results alongside patient imaging in our hospital.

### Fate of detected lung nodules

Three board-certified radiologists with more than 10 years of experience in radiology reviewed all CXRs by consensus to determine whether AI-detected lung nodules were true positive or false positive results. For false positive findings, radiologists searched for the reason for the false positive results using follow-up images and EMRs.

The radiologists categorized the true positive results into four groups: malignancy (group A), active inflammation or infection that needs treatment (group B), post-inflammatory sequelae including granulomas (group C), and others (group D). Nodules that were defined as true nodules on CXR but did not fall into groups A, B, or C were categorized as group D. The clinical outcomes and consequences of nodule detection were reviewed by examining EMRs up to May 2022. For group A, malignancy was determined through pathologic confirmation of the lung nodule itself or clinical diagnosis using follow-up images, including positron-emission tomography (PET)-CT scans. For group B patients, active inflammation or infection was confirmed through bronchoalveolar lavage, sputum culture, or clinical diagnosis using serial CT scans following medication. For group C, post-inflammatory sequelae were noted when the imaging features did not change during follow-up and as determined by a consensus reading of the three radiologists. Group D patients were categorized using CT scans. The reason for CXR, the department of the ordering physician, additional tests or therapeutic intervention for diagnosis and treatment, and final clinical outcomes of detected lung nodules were analyzed as much as possible.

In our hospital, CT images were obtained with a 256-slice CT scanner (Brilliance iCT Elite or IQon Spectral CT; Phillips) according to clinical demands. The CT parameters are tube voltage: 100 kVp; automatic tube current modulation: Dose right; table pitch: 0.6; detector configuration: 128 × 0.625 mm; Gentry rotation time: 0.4; and slice thickness/interval: 1/2 mm.

### Analyzing co-existing lesions on CXR for group categorization

To determine whether co-existing lung abnormalities on CXR detected by AI can help differentiate true positive nodules from false positive results and to categorize groups of true positive nodules, abnormality scores for nodules, atelectasis, consolidation, fibrosis, and pleural effusion were evaluated on CXR.

### Statistical analysis

Statistical analyses were performed using SPSS version 25.0 (IBM Corp., Armonk, NY, United States). Kolmogorov–Smirnov test was performed to determine whether each abnormality score was normally distributed, and values are presented as median with interquartile range (IQR). Mann–Whitney U test was performed to compare abnormality scores between patients with false positive and true positive nodules. Fisher's exact test was performed to compare the rate of nodule malignancy according to co-existing radiologic abnormalities. Kruskal–Wallis test was used to compare abnormality scores among members of groups A, B, and C, while subgroup analyses employed the Donn procedure. To determine whether co-existing lesions indicated the categories of each group with true positive nodules, univariate logistic regression analysis was performed using variables of atelectasis, consolidation, fibrosis, and pleural effusion. In addition, the presence of co-existing abnormalities as an indication of malignancy was evaluated using the logistic regression test. Statistical significance was considered at *P* values < 0.05.

## Results

### Subjects

**Figure 1:**
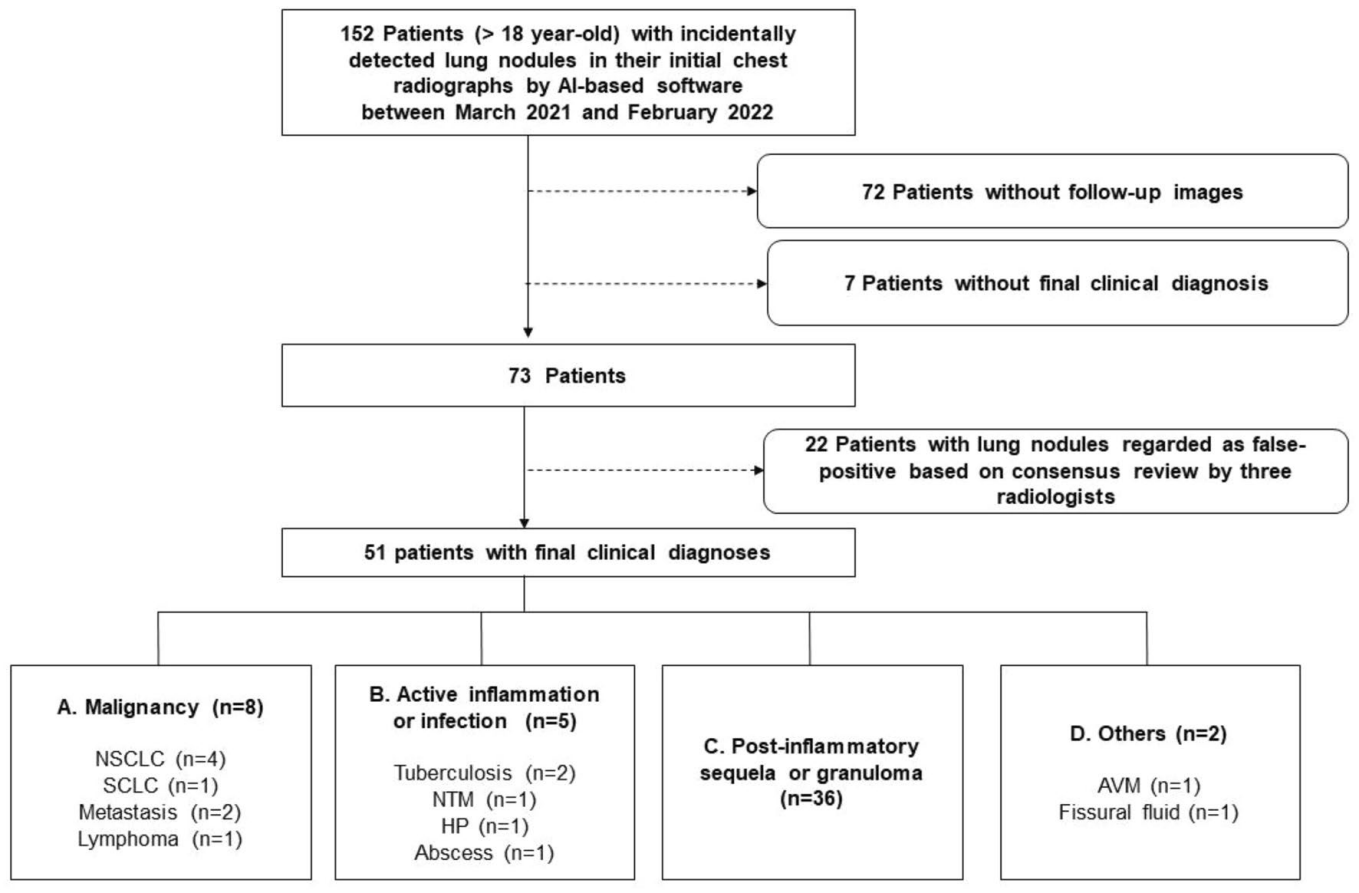
Flow chart of patient inclusion. AI, Artificial intelligence; AMV, arteriovenous malformation; HP, hypersensitivity pneumonitis; NSCLC, non-small cell lung cancer; NTM, nontuberculous Mycobacteria; SCLC, small-cell lung cancer.

During the study period, 14,563 patients underwent initial CXR in outpatient clinics that were not part of the pulmonology or thoracic surgery department. Among them, AI-based software detected unexpected lung nodules in 152 patients (1.0%). Seventy-two patients were excluded due to inconclusive results because they had no follow-up images. In addition, seven patients were excluded because they received no final clinical diagnosis. A total of 73 patients (M:F = 45:28; median age = 70 years, with an age range of 27–90 years) was included in the final analysis. A flowchart of patient inclusion is provided in Fig. 1. The reasons for CXRs were heart evaluation in cardiology (n = 40), preoperative evaluation for general anesthesia (n = 13), health check-up (n = 5), and others with extra-thoracic problems, such as joint pain or renal insufficiency (n = 15).

### Identification of detected lung nodules and clinical outcome

**Figure 2:**
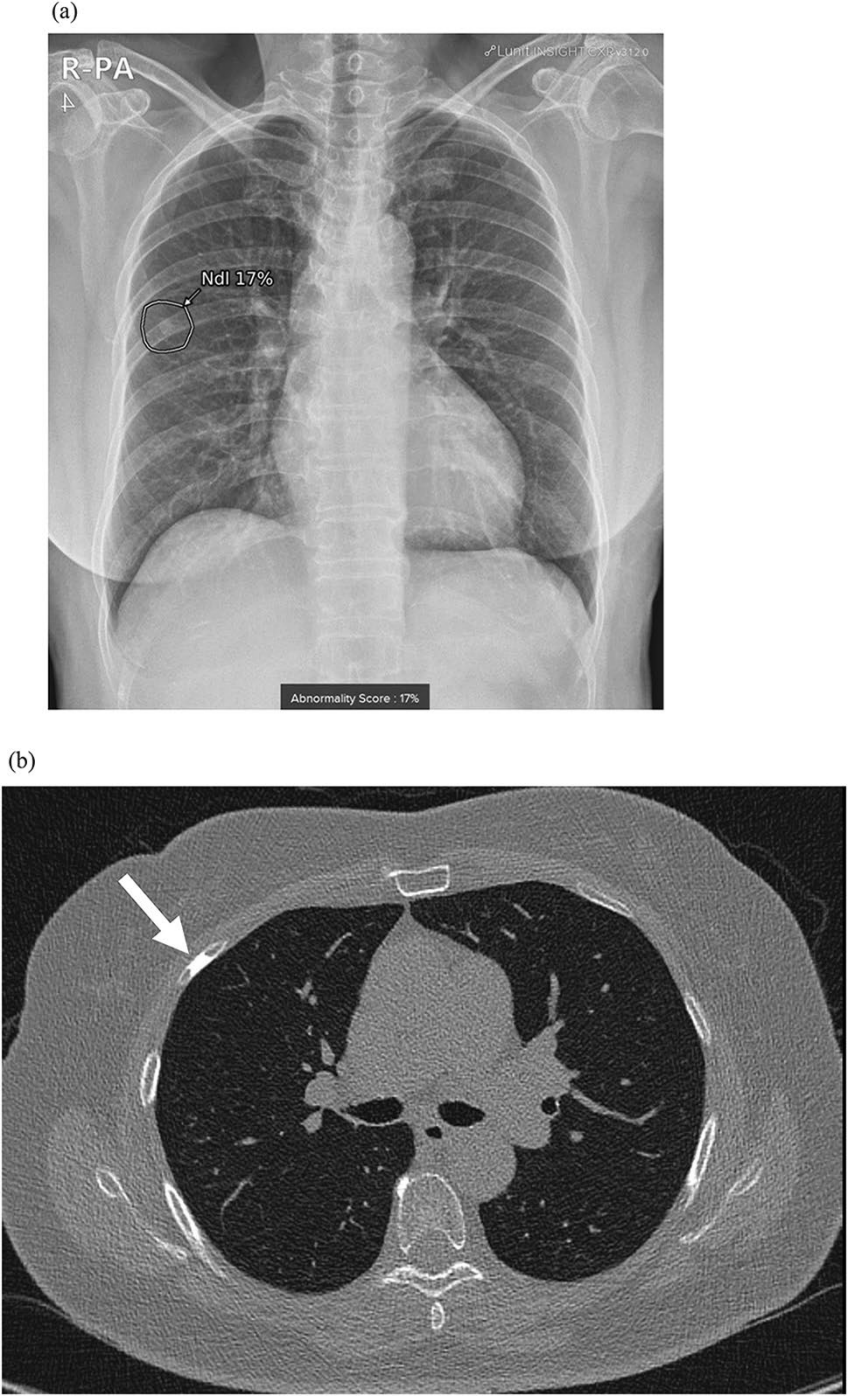
A false positive case of a 71-year-old woman who underwent initial CXR after complaining of headache and hypertension. A nodule detected by AI-software was proven to be a bony island of the right fourth rib. (**a**) AI-software detects a small nodule (arrow) in the right middle lung field with an abnormality score of 17% on CXR. (**b**) Corresponding low-dose CT revealed a small homogeneously sclerotic bone lesion within the medullary space of the right fourth rib (arrow), which would be a bony island.

Among the 73 included patients, 22 (30.1%) were determined to have false positive indications of a detected nodule based on CT (n = 15) or follow-up CXR (n = 7) according to a consensus review. The reasons for false positive findings were no explainable significant lesion (n = 2), bone summation shadow or bony lesions (n = 7, Fig. 2), aorta (n = 2), pulmonary vascular marking or lymph nodes (n = 6), atelectasis (n = 1), pulmonary effusion (n = 3), and pulmonary edema (n = 1).

**Figure 3:**
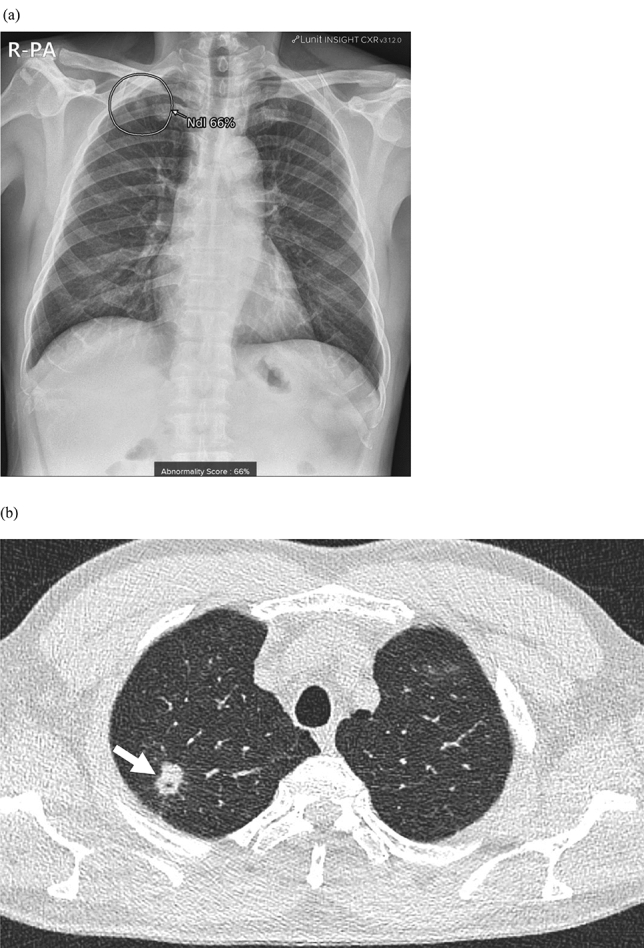
A true positive case of a 64-year-old man in group A with pathologically proven non–small cell lung cancer found in CXR during a health check-up. (**a**) AI-software detected a small lung nodule in the right upper lung with an abnormality score of 66% on CXR. (**b**) A corresponding CT revealed a 1.7-cm non-calcified solid lung nodule with a speculated margin and internal bubble-like air lucencies (arrow). Surgical pathology of right upper lobectomy lesion was adenocarcinoma.

A total of 51 patients (69.9%) showed true positive results (mean size: 27.0 ± 18.7 mm), and eight (11.0%) of whom were included in group A (Fig. 3). Four patients had non–small cell lung cancer, one patient had small-cell lung cancer, two had metastasis from the sigmoid colon or ampulla of Vater cancer, and one had lymphoma involvement in the lung. All were initially diagnosed with CXR. In follow-up of patient management, three patients with non–small cell lung cancer underwent surgery, and the remaining five received chemotherapy for the discovered lung lesions.

**Figure 4:**
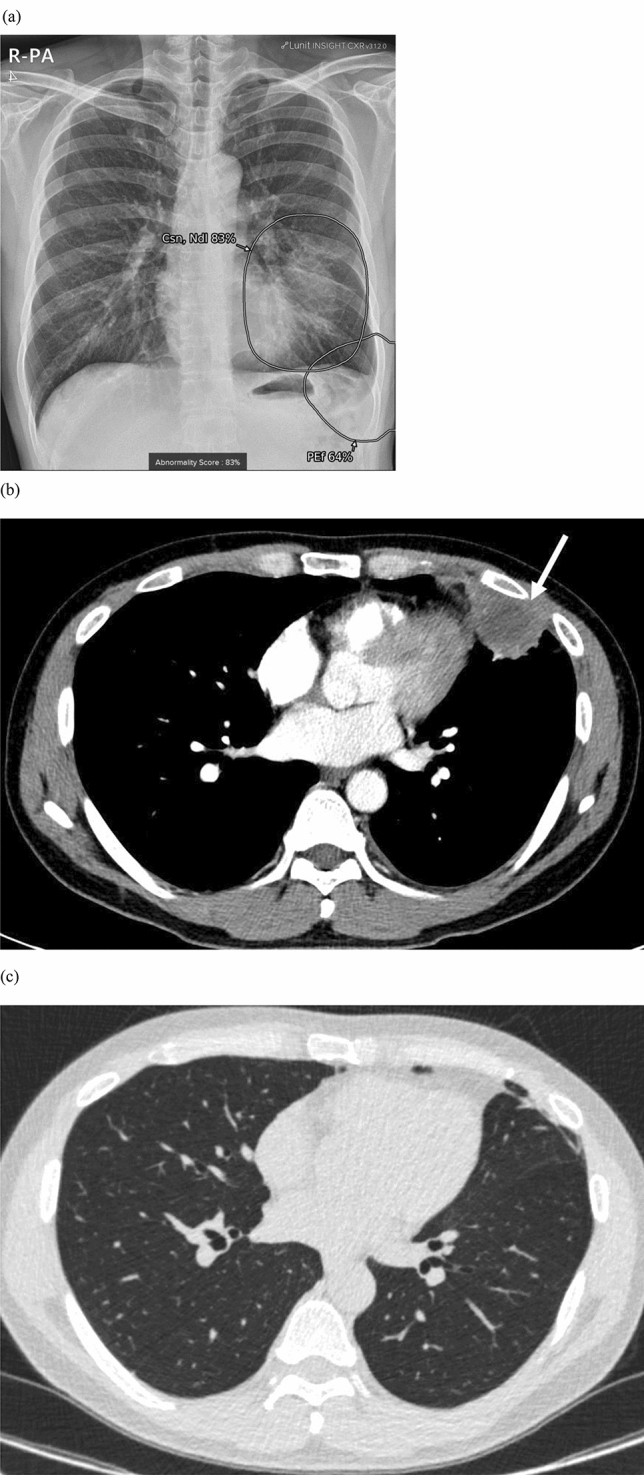
A true positive case from group B with lung abscess and pneumonia in a 47-year-old man who visited a cardiology outpatient clinic for chest pain. (**a**) On CXR, AI-software identified a large, round, mass-like opacity in the left-middle and lower lung zones as a nodule and/or consolidation, with abnormality scores of 79% and 83%, respectively. Pleural effusion was suspected, with an abnormality score of 64%. (**b**) Contrast-enhanced CT revealed an approximately 4-cm necrotic lung mass combined with consolidation in the left lingular segment (arrow) and a small amount of pleural effusion. (**c**) After antibiotic treatment, the lesion decreased greatly, except for residual minimal atelectasis.

Five patients (6.9%) were included in group B (Fig. 4). Two had active pulmonary tuberculosis, one had nontuberculous mycobacteria infection; of them, one was cured after anti-tuberculosis medication for 6 months, and two were transferred to another hospital for treatment. Of the remaining two patients, one had hypersensitive pneumonitis and the other had lung abscess. These lesions improved after treatment with steroid and antibiotics, respectively.

**Figure 5:**
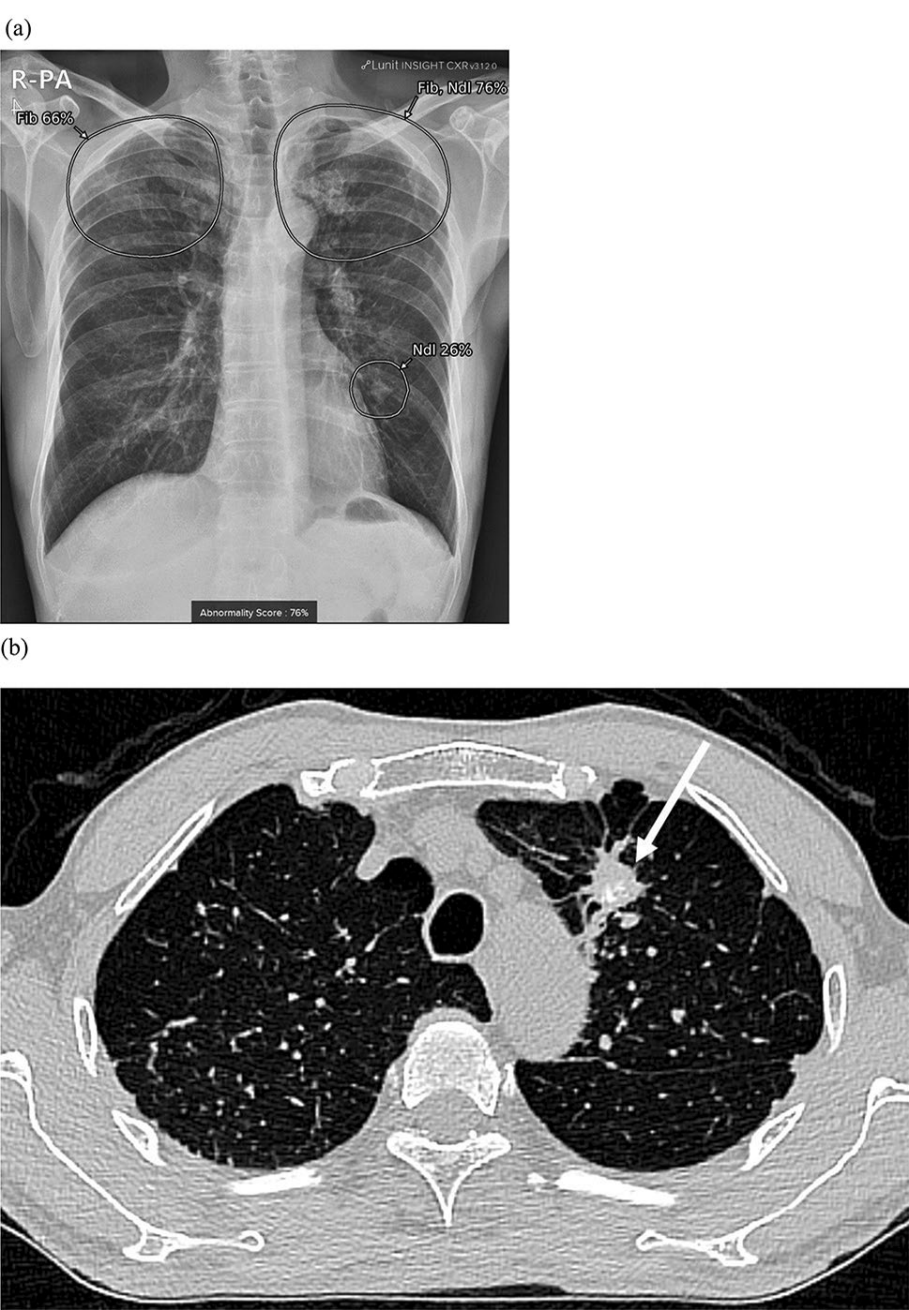
A true positive case in a 72-year-old male in group C with a granuloma on CXR after visiting the neurology outpatient clinic for memory disturbance. (**a**) On CXR, a small lung nodule was detected in the left-middle to lower lung field by AI software, with an abnormality score of 26%. Co-existing fibrosis was suspected in the apex of bilateral upper lungs, with an abnormality score of 76%. (**b**) Contrast-enhanced chest CT revealed a calcified nodule (arrow) with branching fibronodular lesions and architectural distortion in both apical lungs, which suggests post-inflammatory changes.

Group C comprised 36 patients (49.3%) with post-inflammatory sequelae including granulomas (Fig. 5). Group D comprised two patients (2.7%, 2/73), one that was proven to be a pulmonary arteriovenous malformation and one that had fissural fluid mimicking a lung mass. The fissural fluid has resolved without treatment by follow-up CXR.

### Analysis of abnormality scores for group categorization

A comparison of abnormality scores between patients with true positive and false positive nodules is summarized in Table 1. In all patients, the abnormality score for nodules was significantly higher in patients with true positive results compared with those with false positive nodules (median 24.2% vs. 19.3%, *P* = 0.025). The abnormality score for fibrosis was also significantly higher in patients with true positive nodules (median 28.3% vs. 4.3%, *P* = 0.001), while the abnormality scores for atelectasis, consolidation, and pleural effusion were not significantly different.

**Table 1:** Comparison of abnormality scores.

	True positive (n = 51)	False positive (n = 22)	*P* value*	Group A (n = 8)	Group B (n = 5)	Group C (n = 36)	*P* value*			
							Overall	A versus B	A versus C	B versus C
Atelectasis	2.41 (1.03, 14.36)	3.72 (1.12, 18.52)	0.494	1.04 (0.70, 1.61)	11.31 (1.53, 31.52)	2.87 (1.06, 18.39)	0.055			
Consolidation	12.1 (3.24, 45.69)	13.12 (2.53, 25.39)	0.415	8.95 (4.74, 35.81)	68.69 (21.60, 84.56)	11.06 (2.67, 44.47)	0.123			
Fibrosis	28.27 (4.73, 71.48)	4.28 (1.77, 11.70)	**0.001**	3.84 (2.77, 4.77)	28.27 (2.42, 65.97)	49.36 (8.70, 76.38)	**0.002**	0.131	**0.001**	0.317
Nodule	24.18 (18.82, 37.63)	19.25 (15.93, 25.56)	**0.025**	85.15 (37.39, 97.28)	27.21 (20.38, 58.17)	21.65 (16.62, 33.79)	**0.001**	0.142	** < 0.001**	0.234
Pleural effusion	1.45 (0.55, 22.18)	2.69 (0.62, 40.60)	0.533	1.48 (0.44, 52.17)	64.67 (1.50, 97.12)	1.33 (0.56, 12.67)	0.215			

Values are presented as median with interquartile range (IQR). Significant values are in bold.

*Comparison between true and false positive groups: Mann–Whitney U test.

**Comparison between group A-C: Kruskal–Wallis tests with Dunn’s procedure (in subgroup analysis, corrected *P* value < 0.0167 was significant).

Among 73 included patients, the rate of malignancy was higher in patients without co-existing abnormalities compared to patients with co-existing abnormal radiologic findings (19.2% [5/26] vs. 6.4% [3/47]; *P* = 0.124), although the difference was not significant. For 51 patients with true positive nodules, the rate of malignancy was significantly higher in patients without other abnormalities compared to patients with co-existing abnormal radiologic findings (35.7% [5/14] vs. 8.1% [3/37]; *P* = 0.028).

A comparison of abnormality scores among groups A–C is presented in Table 1. Among the patients with true positive nodules, those in group A showed significantly higher abnormality scores for nodules (median 85.2% vs. 21.7%, *P* = 0.001) and lower abnormality scores for fibrosis (median 3.8% vs. 49.4%, *P* = 0.002) compared with patients in group C. No other significant differences were found in the abnormality scores for other lesions among the three groups (Table 1).

Results of logistic regression tests are presented in Table 2 and curves for logistic regression analysis are presented in the Supplementary File. In univariate analysis, an increased abnormality score for nodules was significantly associated with group A patients (odds ratio [OR] = 1.076, 95% confidence interval [CI] 1.032–1.122, *P* = 0.001), while other scores showed no significant association. In group B, increased abnormality scores for consolidation (OR = 1.033, 95% CI 1.002–1.066, *P* = 0.040) and pleural effusion (OR = 1.025; 95% CI 1.001–1.050; *P* = 0.041) were significant for predicting nodules from active infection and inflammation. In addition, abnormality scores for fibrosis (OR = 1.036, 95% CI 1.008–1.066, *P* = 0.013) and nodules (OR = 0.940, 95% CI 0.905–0.976, *P* = 0.001) were significantly associated with group C, reflecting post-inflammatory sequelae.

**Table 2:** Univariate logistic regression analysis of factors associated with each group.

	Group A		Group B		Group C	
	OR (95% CI)	*P* value	OR (95% CI)	*P* value	OR (95% CI)	*P* value
Atelectasis	0.730 (0.428–1.244)	0.248	1.009 (0.970–1.050)	0.656	1.022 (0.977–1.069)	0.334
Consolidation	0.988 (0.959–1.018)	0.443	1.033 (1.002–1.066)	**0.040**	0.990 (0.970–1.011)	0.361
Fibrosis	0.840 (0.666–1.059)	0.139	0.995 (0.967–1.024)	0.740	1.036 (1.008–1.066)	**0.013**
Nodule	1.076 (1.032–1.122)	**0.001**	1.004 (0.968–1.042)	0.825	0.940 (0.905–0.976)	**0.001**
Pleural effusion	0.998 (0.975–1.022)	0.859	1.025 (1.001–1.050)	**0.041**	0.988 (0.970–1.005)	0.166

Significant values are in bold.

CI, Confidence interval; OR, odds ratio.

In groups A–C, absence of co-existing abnormalities on CXR was significantly associated with group A (OR 6.875, 95% CI 1.352–34.965, *P* = 0.020). However, in all 73 patients harboring false positive nodules, there was no significant association between absence of co-existing abnormalities and malignancy (*P* = 0.108).

## Discussion

During the study period, lung nodules were detected incidentally by AI software on CXR in 1.0% of patients (152 of 14,563) who underwent initial CXR at an outpatient clinic other than in the pulmonology or thoracic surgery department. Of the 73 patients included in the final analysis, the false positive rate was 30.1%. The proportions of malignancy, active inflammation, post-inflammatory sequelae, and others were 11%, 6.9%, 49.3%, and 2.7%, respectively, indicating that approximately 20.6% of incidental lung nodules of group A, B and D required further evaluation or treatment. In addition, associated lesions could be the clue to differentiate true positive nodules. For example, associated consolidation and pleural effusion could suggest active inflammation and infection, while associated fibrosis indicates postinflammatory sequelae. An AI-detected isolated lung nodule without associated abnormalities on CXR suggest malignancy.

The performance of computer-aided lung-nodule detection has improved rapidly in recent decades with advances in deep learning. The sensitivity and specificity of lung-nodule detection using AI software in CXR have been reported as 44.1%–95.7% and 71.9%–97.5%, respectively^3,23,24^. Overall performance and sensitivity of AI software standalone are similar or superior to those of physicians and radiologists in detecting lung nodule or lung malignancy^2,4,9,10,13,23–27^. When it was used as an adjunct to doctor judgment, lung-nodule detection improved regardless of reader experience^2,4,9,10,23^. However, some nodules were found only by either a radiologist or AI-based detection^3,23,28^. AI software has not been approved for use alone and is currently positioned as a second reader in lung-nodule detection.

Identification of a larger number of lesions does not always yield greater benefits. In a lung cancer screening cohort study, AI-based detection produced a higher false positive rate compared with radiologists^13^. Lung nodules not detected by humans but discovered using AI software may be of low clinical importance and not require additional workup or treatment, which may lead to unnecessary CT scans. However, a study by Jang et al. of a healthy control group found no significant difference in the rate of unnecessary chest CT recommendations due to false positive detection regardless of AI software use^23^. This suggests that physician judgment is a decisive factor in patient management. When deciding whether to conduct further evaluation, various data such as clinical information of the patient; radio-opacity reflecting lesion calcification, border, or distribution of the lesion; and accompanying additional imaging features such as fibrosis or pleural effusion are considered by physicians. The latest AI-based detection software provides probabilities for various lung abnormalities in addition to nodules. We attempted to determine whether characterization of detected nodules could be aided by additional imaging features on CXR to minimize false positive results and select clinically significant nodules. Abnormality scores for nodule, atelectasis, and fibrosis in group A were significantly different from those of patients in group C. Presence of co-existing abnormalities detected by AI software was correlated with malignancy rate of incidental lung nodules, although combined false positive and group C cases were most common regardless of the presence of other abnormal findings. Using AI information from accompanying CXR abnormalities may be helpful in identifying malignant lesions from lesions with low need for additional evaluation. Even though there is no proven threshold size for lung nodule detection on AI-assisted CXR^29^, one study demonstrated that the discovery of nodules using AI had led to incidental early detection of pulmonary malignancies^30^.

The AI-based software detection of nodules and various image findings on CXR are comparable to or superior to those of radiologists^3,4,24,25^. However, the ability of AI to make differential diagnosis of specific disease entities remains suboptimal (based on a pooled overall accuracy of 0.686), with the exception of pneumothorax diagnoses^2^. Other than identification of image findings, differentiating lung disease entities is a difficult task, and radiologists have difficulties interpreting CXR. This is because various disease entities with different pathologies can result in similar image patterns and overlap in 2D imaging features. In this study, the abnormality score of each image finding tended to match the clinical expectation for each disease group. Such a trend may be helpful in differential diagnosis of disease, but additional research is needed.

There were some limitations in this study. First, the number of patients included in the study is not large. This was unavoidable as data collection has only been possible since the latest versions of AI-based detection software were integrated into daily practice. Second, approximately half of patients with incidentally detected lung nodules were excluded from the final study population due to inconclusive results and lack of a standard reference. We suggest that excluded lesions would have included clinically nonsignificant lesions that, as judged by the clinician, did not require further evaluation and lesions for which the patients refused further evaluation despite significant radiologic findings. Third, as this study targeted nodules with a score of 15% or higher in AI analysis, false negatives could not be identified. Fourth, we could not evaluate the diagnostic performance of AI nodule detection in comparison with the diagnostic abilities of radiologists. Because the aim of this study was to assess the clinical significance of nodules detected by AI and to evaluate how these findings impacted patient treatment, we are planning to address these issues in a subsequent study. Finally, there is a lack of information on detection and management of lung nodules depending on use of AI-based detection software. In our hospital, comparative studies were not possible because clinicians can assess the AI results whenever they want, but we were not able to verify whether the decision-making process of the included patients included reference to AI results. Further research is needed in collaboration with hospitals that have not yet introduced AI-based detection software.

In conclusion, our results showed that lung nodules were detected unexpectedly by AI in approximately 1% of initial CXR, and approximately 70% of these cases were true positive nodules, while 20.5% needed clinical management. The use of AI-based lesion-detection software on CXR in daily practice could help identify clinically significant incidental lung nodules, and referring accompanying lung lesions on CXR may help classify the nodules.

### Supplementary Information

Supplementary Figures.

## Data Availability

The datasets generated and analyzed during the current study are available from the corresponding author on reasonable request.
