# Supplementary material for: Clinical outcomes and actual consequence of lung nodules incidentally detected on chest radiographs by artificial intelligence
Source: Sci Rep. 2023 Nov 13;13:19732. doi: 10.1038/s41598-023-47194-6 (PMC10643548; doi:10.1038/s41598-023-47194-6)
Supplement: Supplementary file 1 — Supplementary Figures. [file 41598_2023_47194_MOESM1_ESM.pdf]

Supplementary file.

Supplementary Figures S1. Receiver operating characteristic (ROC) curve for univariate logistic regression analysis of factors associated with group A. (a) Atelectasis, (b) Consolidation, (c) Fibrosis, (d) Nodule, (e) pleural effusion.

(a)

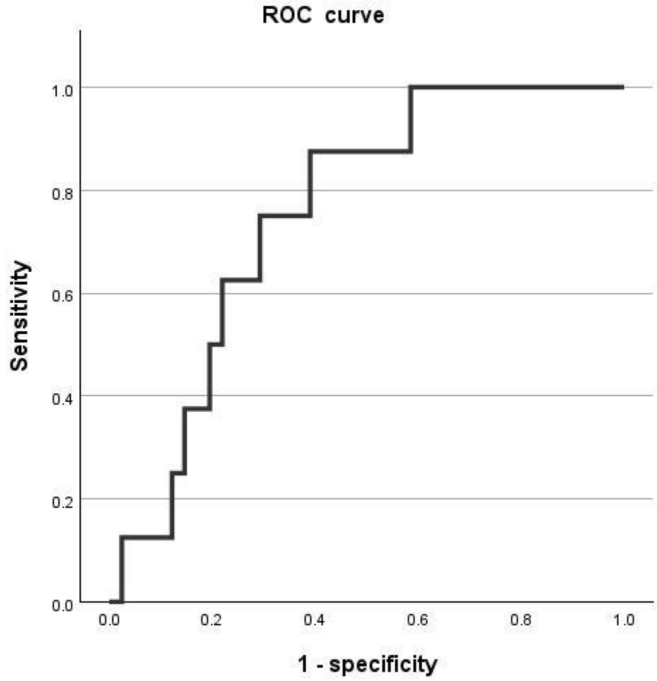

(b)

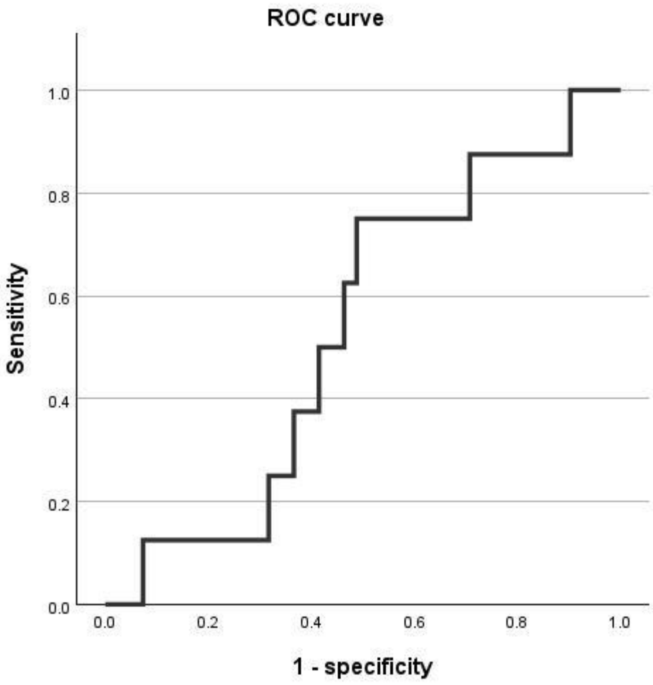
ROC curve
Sensitivity
1.0
0.8
0.6
0.4
0.2
0.0
0.0
0.2
0.4
0.6
0.8
1.0
1 - specificity

(c)

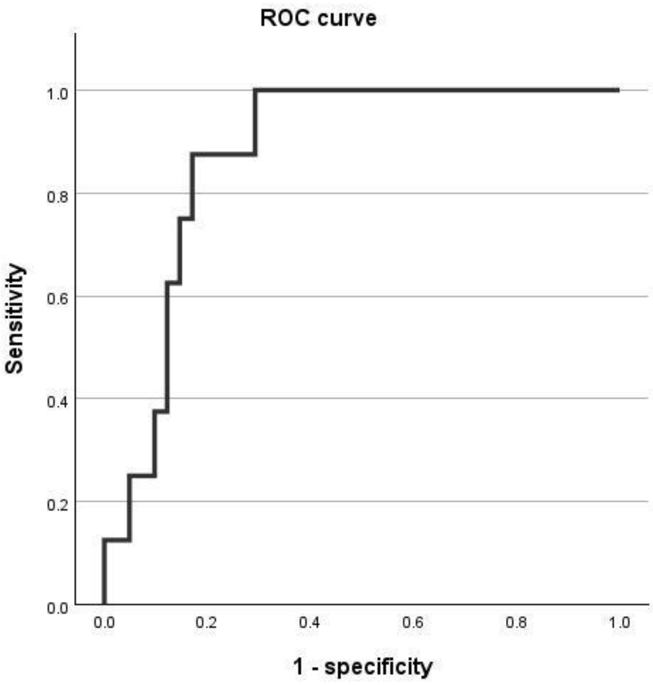
ROC curve
Sensitivity
1.0
0.8
0.6
0.4
0.2
0.0
0.0
0.2
0.4
0.6
0.8
1.0
1 - specificity

(d)

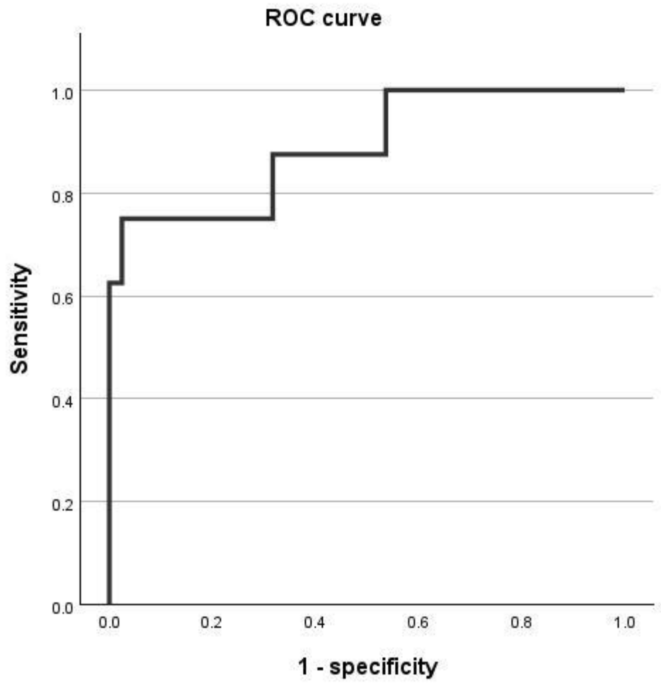
ROC curve
Sensitivity
1.0
0.8
0.6
0.4
0.2
0.0
0.0
0.2
0.4
0.6
0.8
1.0
1 - specificity

(e)

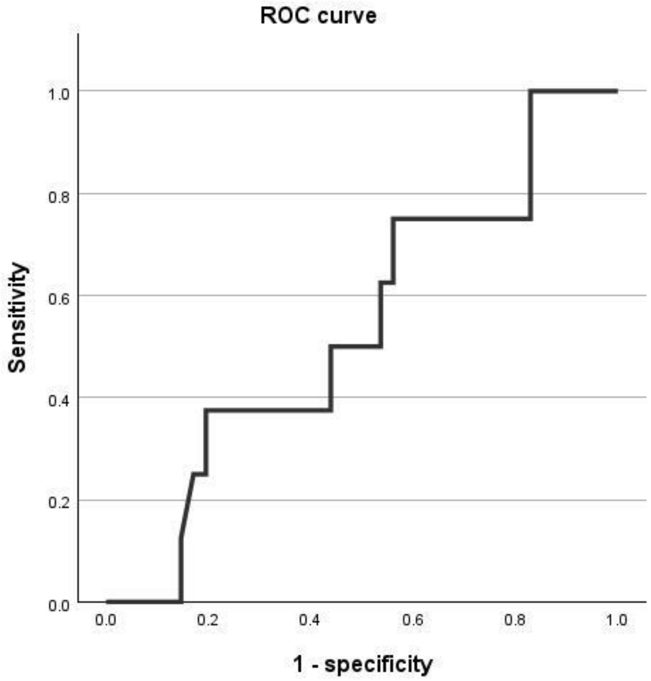

ROC curve
Sensitivity
1.0
0.8
0.6
0.4
0.2
0.0
0.0
0.2
0.4
0.6
0.8
1.0
1 - specificity

Supplementary Figures S2. ROC curves for univariate logistic regression analysis of factors associated with group B. (a) Atelectasis, (b) Consolidation, (c) Fibrosis, (d) Nodule, (e) pleural effusion.

(a)

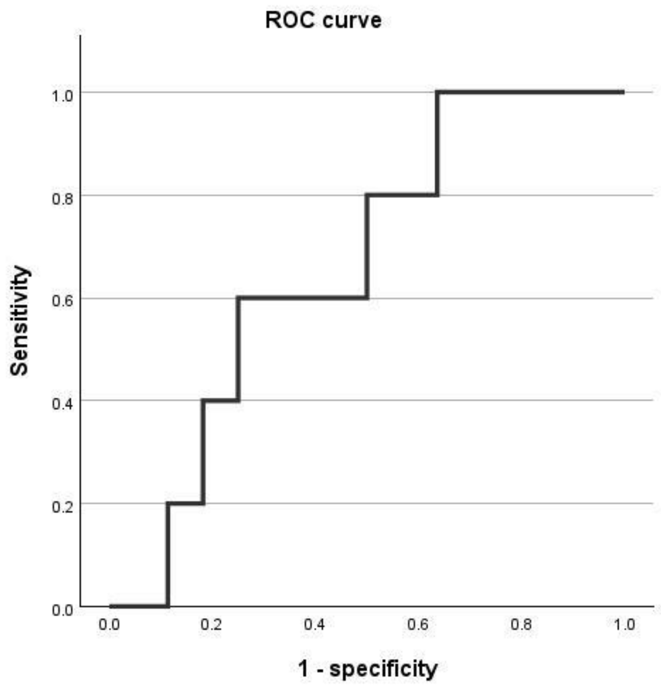

(b)

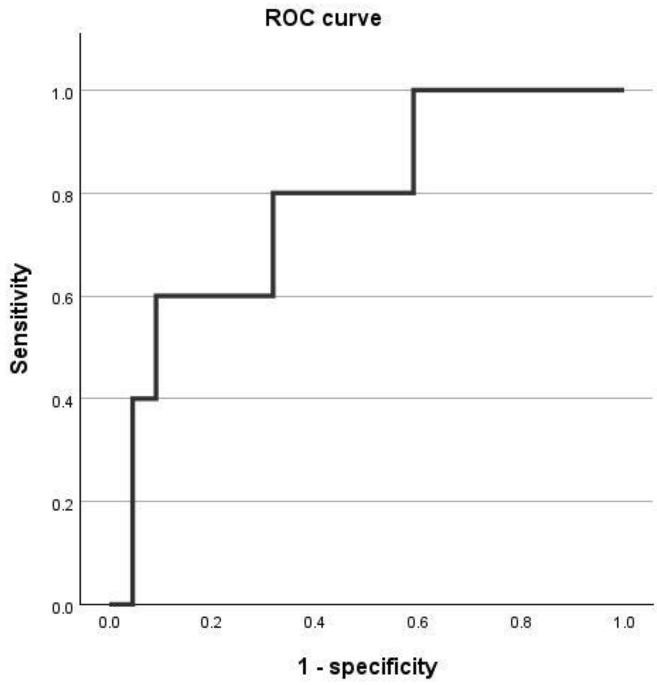
ROC curve
Sensitivity
1 - specificity
0.0
0.2
0.4
0.6
0.8
1.0

(c)

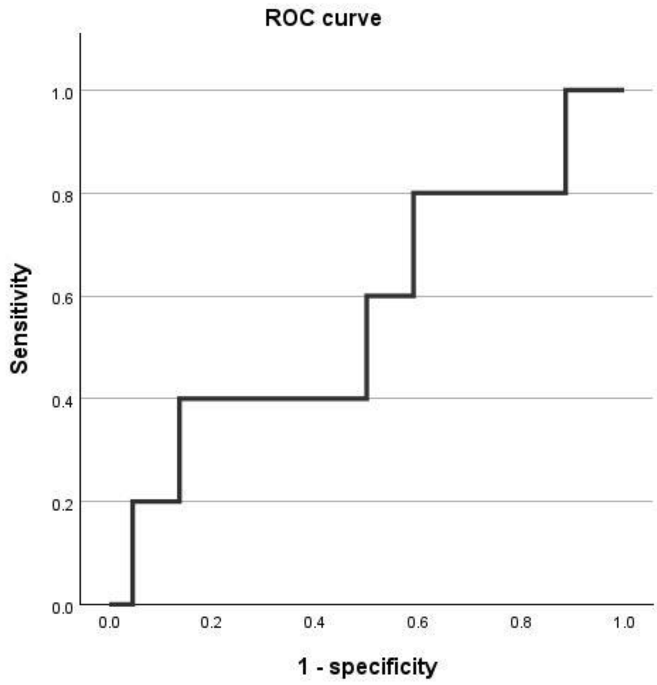
ROC curve
Sensitivity
1 - specificity
0.0
0.2
0.4
0.6
0.8
1.0

(d)

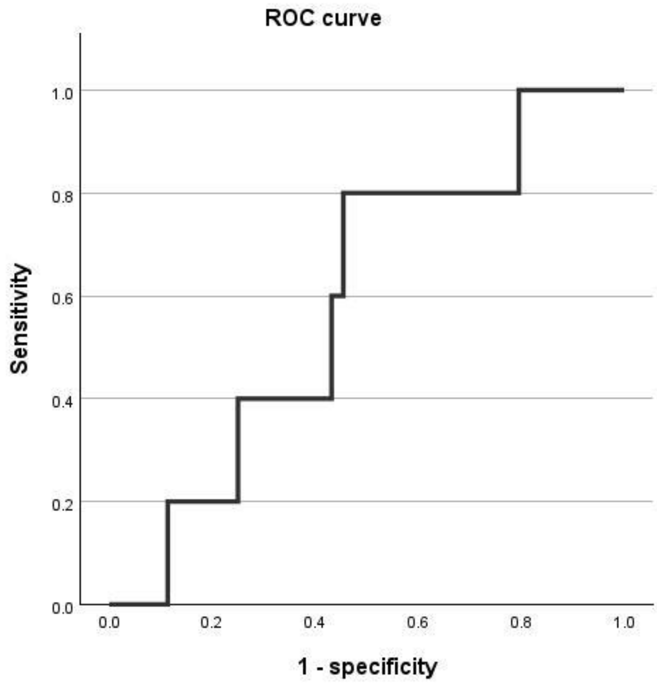
ROC curve
Sensitivity
1.0
0.8
0.6
0.4
0.2
0.0
0.0
0.2
0.4
0.6
0.8
1.0
1 - specificity

(e)

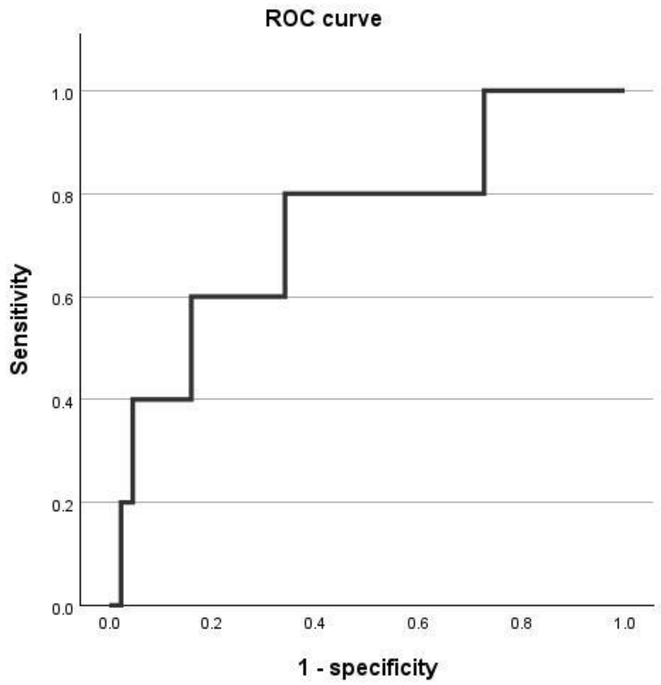
ROC curve
Sensitivity
1.0
0.8
0.6
0.4
0.2
0.0
0.0
0.2
0.4
0.6
0.8
1.0
1 - specificity

Supplementary Figures S3. ROC curve for univariate logistic regression analysis of factors associated with group C. (a) Atelectasis, (b) Consolidation, (c) Fibrosis, (d) Nodule, (e) pleural effusion.

(a)

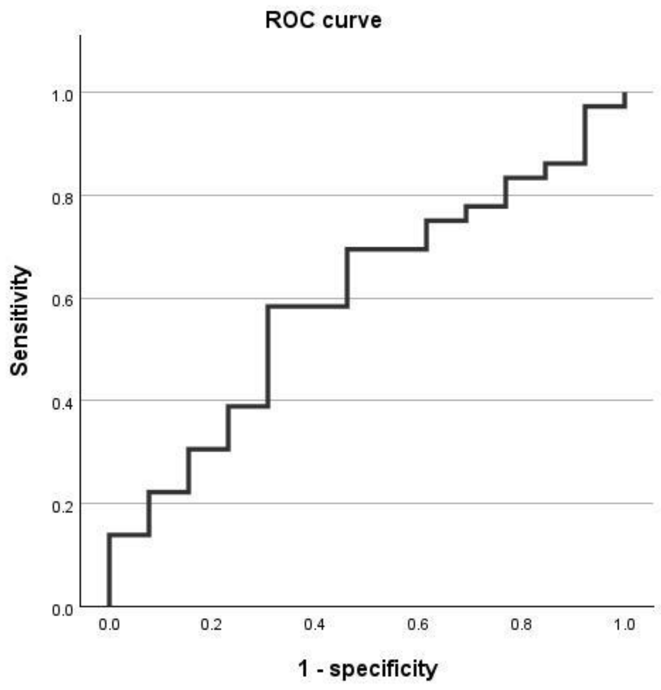

(b)

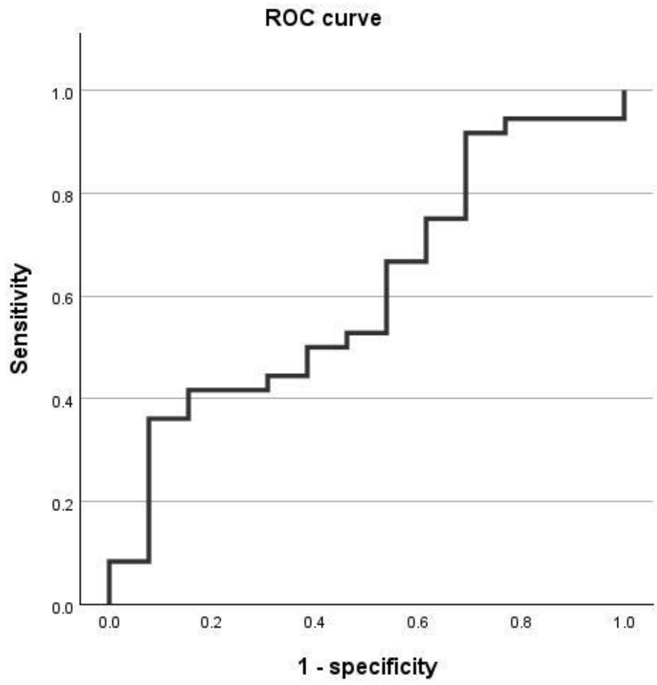
ROC curve
Sensitivity
1 - specificity
0.0
0.2
0.4
0.6
0.8
1.0

(c)

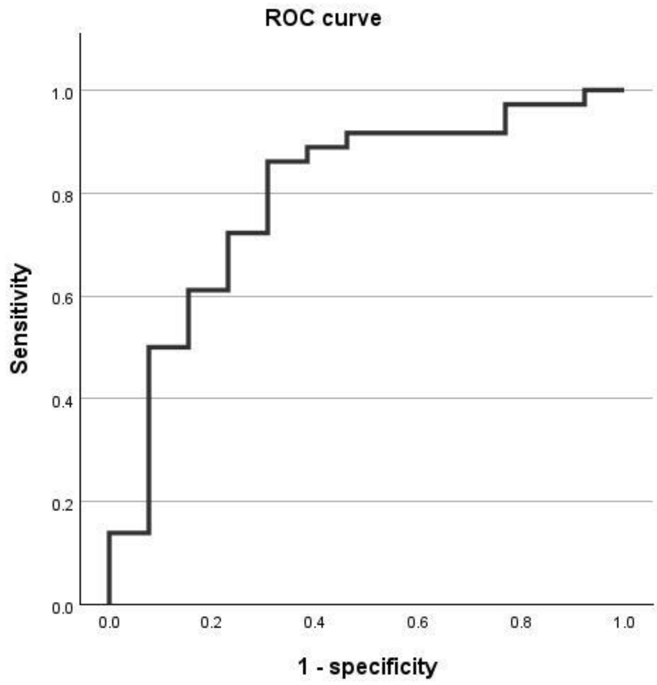
ROC curve
Sensitivity
1 - specificity
0.0
0.2
0.4
0.6
0.8
1.0

(d)

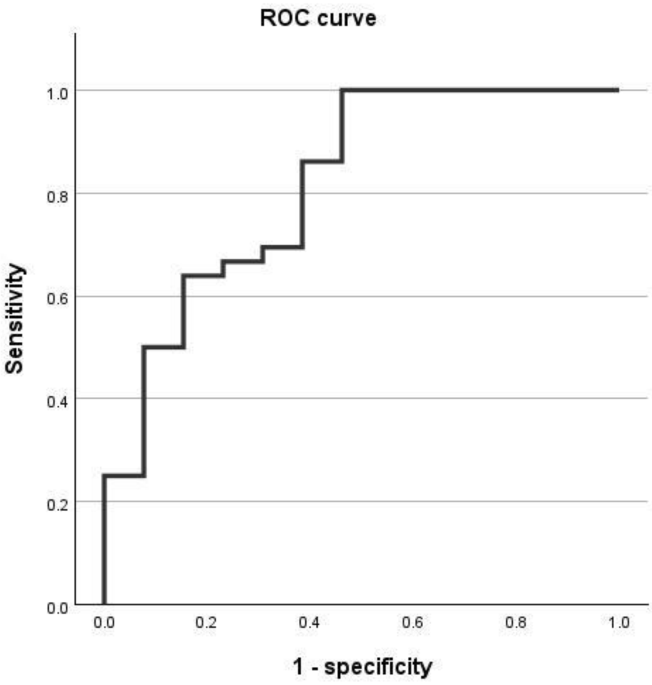

ROC curve
Sensitivity
1 - specificity
0.0
0.2
0.4
0.6
0.8
1.0

(e)

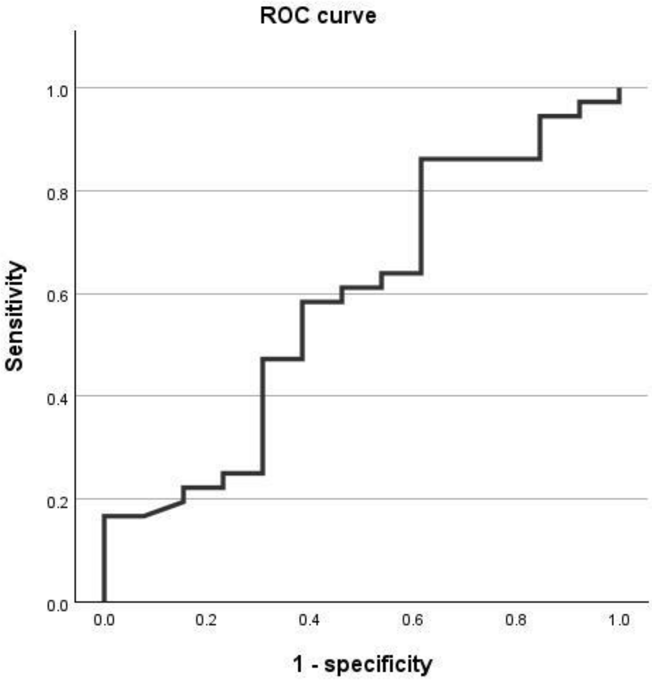
ROC curve
Sensitivity
1.0
0.8
0.6
0.4
0.2
0.0
0.0
0.2
0.4
0.6
0.8
1.0
1 - specificity
